# Adnexal Torsion during Pregnancy after Oocyte In Vitro Maturation and Intracytoplasmic Sperm Injection Cycle

**DOI:** 10.1155/2010/141875

**Published:** 2010-08-16

**Authors:** Simone Giulini, Giulia Dante, Susanna Xella, Antonio La Marca, Tiziana Marsella, Annibale Volpe

**Affiliations:** Mother-Infant Department, Center of Reproductive Medicine, Institute of Obstetrics and Gynecology, University of Modena and Reggio Emilia, University Hospital of Modena, L.go del Pozzo 71, 41100 Modena, Italy

## Abstract

We report a case of right adnexal torsion during pregnancy after an oocyte in vitro maturation and intracitoplasmic sperm injection cycle in patient with polycystic ovary syndrome. A 31-year-old woman with a typical clinical disorder of polycystic ovarian syndrome was included in an oocyte in vitro maturation program. Right adnexal torsion occurred two days after embryo transfer, and laparoscopy detorsion was successfully performed with preservation of adnexa. The patient had a full-term pregnancy and delivered a healthy infant at 40 weeks of gestation. To our knowledge this is the first report of adnexal torsion after an oocyte in vitro maturation and intracitoplasmic sperm injection program.

## 1. Introduction

After two decades of complex fertility treatment regimens with high doses of hormones, simpler and more physiological protocols have been implemented in several IVF centers. The most recent clinical application in ART has been oocyte in vitro maturation (IVM) [1]. IVM involves the growth of immature oocytes (germinal vesicle stage) in culture up to the metaphase II stage after their earlier retrieval from the ovaries. 

 Adnexal torsion is an emergency condition where the adnexa rotate on their pedicle compromising their blood supply. It is a rare cause of acute abdominal pain and a true gynaecologic emergency. It accounts for approximately 3% of gynaecological emergencies, and 10–20% of ovarian torsions occur during pregnancy, frequently occurring in the first trimester after ovarian stimulation for IVF [2–5]. 

 Here, we report a case of unilateral adnexal torsion during pregnancy after an oocyte in vitro maturation cycle. To our knowledge, this is the first report of adnexal torsion in an oocyte in vitro maturation program.

## 2. Case Presentation

**Figure 1:**
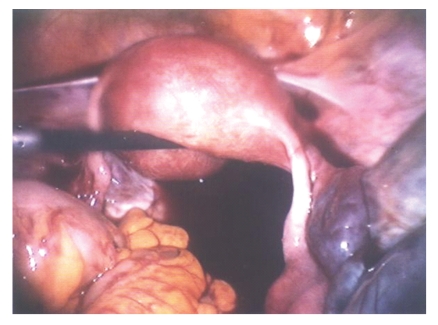
Laparoscopic photograph of right adnexal torsion at the utero-ovarian pedicle.

A 31-year-old healthy woman with a history of primary infertility for 4 years was referred to our Center of Reproductive Medicine and Assisted Procreation in May 2008. The patient had the typical clinical and echographic criteria of polycystic ovarian syndrome (PCOS) [6]. Furthermore, in 2004, a diagnostic laparoscopy revealed patent tubes but with a tortuous course and a mildly dilated left fallopian tube. The male partner had normal semen parameters according to World Health Organization (WHO) standards [7]. In 2006, in another infertility center, the patient underwent two cycles of ovulation induction with intrauterine insemination without achieving pregnancy. In September 2008, at our center, the couple was included in an In Vitro Fertilization (IVF) program. Controlled ovarian hyperstimulation (COH) was performed using a flexible GnRH antagonist protocol with mild stimulation that involved the administration of recombinant FSH (rFSH) (Gonal-F; Serono, Geneva, Switzerland), 150 UI daily, from day five of spontaneous menstrual cycle. During ultrasound monitoring, when at least one follicle reached 14 mm in diameter, GnRH antagonist (Orgalutran 0.25 mg; Organon, Italia) 0.25 mg/day was added subcutaneously. After 9 days of administration of recombinant FSH, controlled ovarian stimulation was suspended for an increased risk of ovarian hyperstimulation syndrome (OHSS). After 2 months, the patient was enrolled in an oocyte vitro maturation (IVM) program. The patient received an oral contraceptive to induce menstruation, then an FSH “priming” was achieved with the administration of recombinant FSH (rFSH) (Gonal-F; Serono, Geneva, Switzerland), 150 UI daily for 3 days (from day three to day five). At day 8, transvaginal ultrasound monitoring showed right and left ovarian measurements of 4.3 × 3.1 cm and 3.9 × 2.7 cm, respectively, with several follicles in both ovaries, ranging from 7 to 11 mm in diameter. The day after recombinant chorionic gonadotropin 250 mcg (Ovidrel; Serono, Geneva, Switzerland) was administered. Thirty-six hours later, transvaginal oocyte retrieval was performed. After collection, the follicular fluid was filtered through a 70 *μ*m cell strainer (Nunc) and was washed twice with flushing medium containing heparin (MediCult, Jyllinge, Denmark). At least 18 cumulus oocyte complex (COC) were collected. After washing all, COCs were placed in a single-well Petri dish (Becton-Dickinson), containing 0.5 ml of pre-equilibrated IVM medium (vial1- LAG 1 of IVM system medium; Medicult, Jyllinge, Denmark) for 3h and then placed in a new single-well Petri dish (Becton-Dickinson) containing 0.5 ml of pre-equilibrated IVM medium (vial 2-LAG 2 of IVM system medium; Medicult, Jyllinge, Denmark) supplemented with 0.075 IU/ml recombinant FSH (Gonal-F; Serono, Geneva, Switzerland), 0.1 IU/ml rHCG (Ovidrel; Serono, Geneva, Switzerland), and 10% maternal serum, inactivated at 56°C. The immature oocytes were stored in an incubator at 37°C and 5% CO_2_in a humidified atmosphere for 30 hours. After 30 hours of culture, all the oocytes were treated with 80 IU/ml hyaluronidase solution in order to remove the cumulus complex, then detected under a stereomicroscope. At least 14 oocytes to 18 reached the MII stage. According to cytoplasmic characteristics and presence of meiotic spindle signal, 12 oocytes were classified with good quality and those showing signs of mechanical damage or atresia were discarded. Unfortunately, as already worldwide known, the restriction of the Italian law, at the time of this report, allows to inseminate a maximum of three oocytes, while the resulting 9 good quality oocytes have been cryopreserved. The semen sample with normal sperm parameters was prepared as previously described [8]. Although normal results of the semen parameters we use ICSI for insemination and all 3 oocytes showed fertilization. Fertilization was assessed 16–18 hours after injection by the presence of two pronuclei. All the resulting zygotes were cultured in ISM1 medium as previously described [8]. The embryos were cultured for 2 days. Three good quality embryos, were transferred on day 3 after oocyte retrieval, as determined by Italian law. For luteal phase support, Progesterone 400 mg (Progeffik 200 mg; Effik, Italy) daily vaginal administration was commenced. Two days after embryo transfer, the patient presented to our clinic complaining of lower abdominal pain. She was afebrile and physical examination showed a point of maximal tenderness in the right lower abdominal quadrant. There was no vaginal bleeding nor any bowel symptoms, acute appendicitis and renal colic were excluded. The laboratory workup showed a white blood cell count of 22.000 migl./ml whereas hepatic enzymes, hematocrit and urine analysis were normal. Transvaginal ultrasound was carried out showing enlarged (10 × 7.5 cm) right ovary within coexistent mass, a small amount of fluid was revealed in the pouch of Douglas. The Colour Doppler examination was normal with presence of ovarian vascular flow bilaterally. Six hours after admission to the hospital, a worsening of clinical symptoms showed an acute abdominal pain. In view of these findings an explorative laparoscopy was carried out. The laparoscopic findings showed a twisted right adnexa whit an ischemic ovary. The enlarged cystic ovary had a bluish hue and measured about 10 cm in diameter. The surgeons proceeded to underwinding the twisted adnexa, pushing the ovary in the opposite direction of the torsion (Figure 1). After a few minutes, there was recoloration and a decrease of the adnexal edema, signs of a successful recovery, and the ovary was preserved. The patient had an unremarkable postoperative course and was discharged to home the following day. Four weeks later transvaginal ultrasound showed a normal intrauterine 6-week pregnancy. The patient had a full-term pregnancy and delivered a healthy infant at 40 weeks of gestation. 

 To our knowledge, this is the first report of adnexal torsion after an IVM cycle.

## 3. Discussion

The most recent clinical application in ART has been IVM. IVM can be a potentially useful intervention for women with PCOS-related infertility since these oocytes can retain their maturational and developmental competence [9]. It is believed that women with PCOS who are at risk of developing OHSS following a conventional controlled ovarian hyperstimulation regimen might benefit from earlier retrieval of oocytes followed by IVM, thus reducing the risk of OHSS. On the other hand, the process of IVM could affect the quality of oocytes as any intervention in their growth phase would affect oocyte maturation, fertilisation, and subsequent embryo development [10]. The clinical outcomes of IVM have continued to improve after the modest results of the previous studies. The average pregnancy rates in women with PCOS have been reported to be between 22 and 30% [11, 12]. However, there are no adequately controlled studies available [1]. 

 Adnexal torsion is an emergency condition where the adnexa rotate on their pedicle. Torsion of the right adnexa is more common, and few reports of bilateral torsion, either simultaneously or subsequently, exist (13–15). Adnexal torsion frequently occur in the first trimester after ovarian stimulation for IVF (2–5). The diagnosis of ovarian torsion is difficult and occasionally remains a diagnostic dilemma. The clinical symptoms could be confused with other acute abdominal conditions. The signs of adnexal torsion include a palpable pelvic mass, signs of localized peritoneal irritation, a low grade fever, and leukocytosis. Preoperative diagnosis is difficult, especially in pregnant women. When complete torsion with hemorrhagic necrosis is suspected, immediate surgery is necessary. Ultrasonography is the primary imaging modality for evaluation of ovarian torsion. Ultrasonography features of ovarian torsion include a unilateral enlarged ovary, uniform peripheral cystic structures, a coexistent mass within the affected ovary, free pelvic fluid, lack of arterial or venous flow, and a twisted vascular pedicle. The presence of flow at Colour Doppler imaging does not allow exclusion of torsion but instead suggest that the ovary may be viable, especially if flow is present centrally. Absence of flow in twisted vascular pedicle may indicate that the ovary is not viable [13]. Doppler sonography, although highly specific, has low sensitivity, as it may miss the diagnosis in approximately 60% of cases [14]. Adnexal torsion is a surgical emergency during pregnancy. In the past, the traditional treatment was adnexal removal. Nowadays cases of adnexal torsion occurring during the first trimester of pregnancy should preferably undergo laparoscopy, which is suitable for diagnosis, evaluation and treatment [15, 16]. The main specific complications of laparoscopy during pregnancy are related to possible injury to the enlarged uterus and ovaries situated outside the pelvis and to the cardiovascular and respiratory alterations introduced by the pneumoperitoneum pressure and CO_2_ absorption. After laparoscopic detorsion, 24 hours of postoperative observation is recommended. 

 In conclusion, to our knowledge this is the first paper in literature of ovarian torsion of an IVM cycle in pregnant women. Fortunately, this case had a complete resolution with the birth of a healthy infant.
